# A Real-Time and Closed-Loop Control Algorithm for Cascaded Multilevel Inverter Based on Artificial Neural Network

**DOI:** 10.1155/2014/508163

**Published:** 2014-03-16

**Authors:** Libing Wang, Chengxiong Mao, Dan Wang, Jiming Lu, Junfeng Zhang, Xun Chen

**Affiliations:** ^1^State Key Laboratory of Advanced Electromagnetic Engineering and Technology, Huazhong University of Science and Technology, Wuhan 430074, China; ^2^School of Electrical & Electronic Engineering, Huazhong University of Science and Technology, Wuhan 430074, China; ^3^Electric Power Research Institute of Guangdong Power Grid Corporation, Guangzhou, Guangdong 510080, China

## Abstract

In order to control the cascaded H-bridges (CHB) converter with staircase modulation strategy in a real-time manner, a real-time and closed-loop control algorithm based on artificial neural network (ANN) for three-phase CHB converter is proposed in this paper. It costs little computation time and memory. It has two steps. In the first step, hierarchical particle swarm optimizer with time-varying acceleration coefficient (HPSO-TVAC) algorithm is employed to minimize the total harmonic distortion (THD) and generate the optimal switching angles offline. In the second step, part of optimal switching angles are used to train an ANN and the well-designed ANN can generate optimal switching angles in a real-time manner. Compared with previous real-time algorithm, the proposed algorithm is suitable for a wider range of modulation index and results in a smaller THD and a lower calculation time. Furthermore, the well-designed ANN is embedded into a closed-loop control algorithm for CHB converter with variable direct voltage (DC) sources. Simulation results demonstrate that the proposed closed-loop control algorithm is able to quickly stabilize load voltage and minimize the line current's THD (<5%) when subjecting the DC sources disturbance or load disturbance. In real design stage, a switching angle pulse generation scheme is proposed and experiment results verify its correctness.

## 1. Introduction

Multilevel converters have drawn tremendous research interest in recent years and have been implemented in several high-voltage and high-power applications. One of the multilevel topologies is cascaded H-bridges (CHB) configuration which needs several separated DC sources [1].

Various modulation strategies have been developed for CHB converter. Phase shifted PWM (PSPWM) and space vector PWM (SVPWM) are very popular methods in industrial applications [2]. Another important modulation method for CHB converter is the optimal PWM, which includes selective harmonic elimination PWM (SHE-PWM) and harmonic minimization strategies [3]. The main challenge associated with SHE-PWM technique is that a specified number of nonlinear equations must be solved to obtain the appropriate switching angles. Several analytical algorithms have been reported to solve these transcendental equations, such as iterative methods [4], theory of symmetric polynomials [5], and search optimization [6].

The harmonic pollution minimization in multilevel converter with staircase modulation strategy is often defined as a time-limited optimization problem in real-time applications. Modern evolution algorithms have also been used to determine the optimal switching pulses such as genetic algorithms (GA), particle swarm optimization (PSO) [7–9], shuffled-frog-leaping algorithm (SFLA), and Bee algorithm (BA) [10, 11]. In [12], a generalized formulation for multilevel SHE-PWM converters for any number of levels and any number of switching angles with both equal and unequal DC voltage levels was reported. The limitation of quarter-wave symmetrical waveform was relaxed, and half-wave [13] and nonsymmetrical waveforms [14] have also been reported. These papers assumed that the DC source levels do not vary with time. However, the DC voltage levels may vary with time in practice, and calculations methods based on this assumption are very time-consuming. They can only be done by a computer offline. The offline calculated optimal switching angles have to be stored in a look-up table. For every solution in each possible DC voltage level case, a considerable large look-up table would be required. Therefore, the above methods cannot be implemented in real-time manner due to the overhead of computations.

In [15, 16], the authors developed a real-time algorithm to calculate the optimal switching angles. Although the resulted voltage THD is minimized, this iterative algorithm was complex. An alternate approach in which artificial neural network (ANN) is implemented was proposed to replace the look-up table and generate optimal switching angles [17]. However, this method still requires large memory to store numerous solutions and only open-loop control of CHB converter was considered.

In order to real-time-control the CHB converter with variable DC sources under staircase modulation strategy, a real-time and closed-loop control algorithm based on ANN for three-phase CHB converter is proposed in this paper. It needs little computation time and memory and has a good control capability of CHB converter. It has two steps. In the first step, HPSO-TVAC algorithm is employed to calculate the optimal switching angles offline. In second step, only 40 samples of optimal switching angles are used to train an ANN and design an ANN to generate optimal switching angles in a real-time manner. The designed ANN is easily embedded into the proposed closed-loop control algorithm. Simulation results demonstrate that the proposed closed-loop control algorithm can quickly stabilize load voltage and minimize the line current's THD (<5%) subjecting to DC sources disturbance and load disturbance.

The rest of the paper is arranged as follows. In Section 2, harmonic distortion minimization problem of CHB converter is described. In Section 3, the principle of HPSO-TVAC is introduced and compared with other traditional evolution optimization algorithms. In Section 4, ANN is introduced and designed. The proposed ANN is also compared with previous real-time optimization method. In Section 5, a closed-loop control algorithm for CHB converter is proposed, and the simulation results are given to demonstrate the performance of the proposed method in the presence of DC sources disturbance and load disturbance. In addition, the switching angle pulse generation issue is considered in real design stage. Finally, Section 6 concludes the paper.

## 2. Harmonic Minimization Problem 

**Figure 1:**
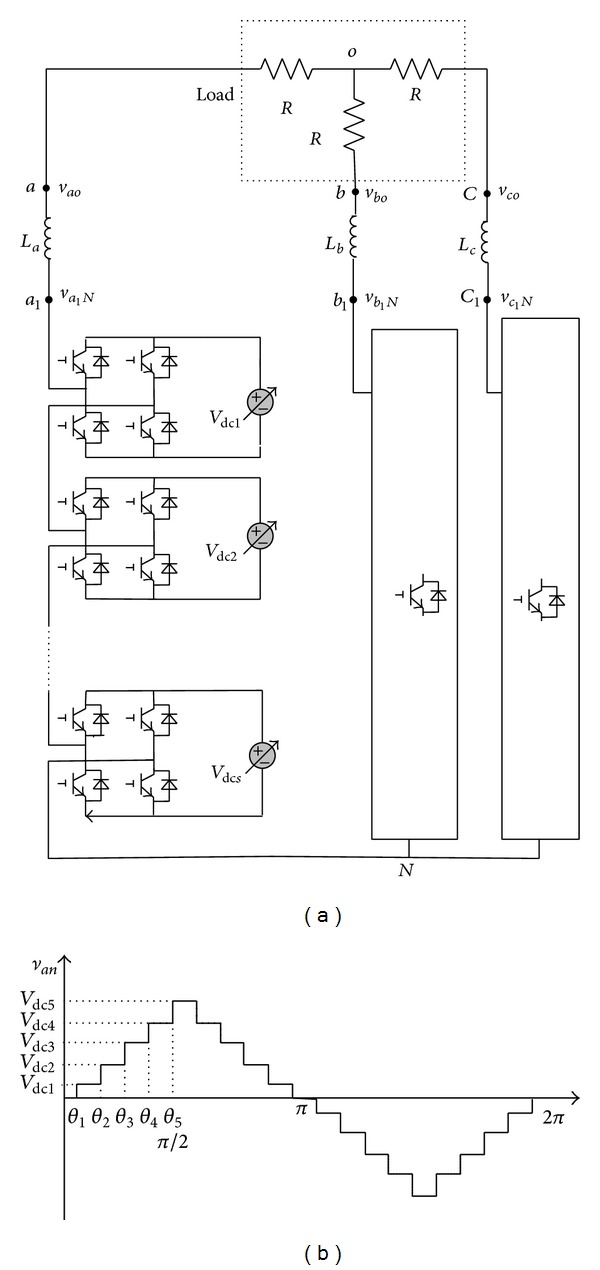
(a) The structure of a three-phase CHB inverter and (b) output phase voltage  *v*_an_ of inverter based on the staircase modulation strategy.

Figure 1(a) shows the structure of a three-phase CHB converter. Each unit has its own separated DC source with a variable voltage level.  Figure 1(b) also shows that the ac terminal output phase voltage is synthesized by the sum of H-bridge voltages; that is, *v*_an_ = *v*_dc1_ + *v*_dc2_ + ⋯+*v*_dc*s*_. Each full bridge can generate three different voltage levels: *v*_dc_, 0, and −*v*_dc_. The number of the levels *m* of output phase voltage in this topology is *m* = 2*s* + 1, where *s* is the number of the DC sources. In the half-cycle waveform, the switching angles set (*θ*_1_, *θ*_2_,…, *θ*_*s*_) is symmetrically arranged. The next half-cycle waveform is similar, but with a negative sign.

The Fourier series expansions of the generated output phase voltage *v*_an_ are

(1) $$\begin{array}{c} v_{\text{an}}=\overset{\infty }{\underset{n=1}{\sum }}\left(a_{n}\sin n\alpha_{n}+b_{n}\cos n\alpha_{n}\right). \end{array}$$

Owing to the quarter-wave symmetry of phase voltage, only odd harmonics are presented in *v*_an_  (*b*_*n*_ = 0). The amplitude of the *n*th harmonic *a*_*n*_ is expressed only with the first quadrant switching angles  *θ*_1_, *θ*_2_,…, *θ*_*s*_

(2) $$\begin{array}{c} a_{n}=\frac{4V_{\text{dc}}}{n\pi }\overset{s}{\underset{k=1}{\sum }}\cos\left(n\theta_{k}\right),\\ 0<\theta_{1}<\theta_{2}<\cdots <\theta_{s}<\frac{\pi }{2}, \end{array}$$

where  *s*  is the number of variables corresponding to switching angles  *θ*_1_ ~ *θ*_*s*_  of the first quadrant; *V*_dc_ is the rated DC voltage level.

In the traditional SHE-PWM method,  *a*_*n*_ was assigned as the desired value for fundamental component and equated to zero for the harmonics to be eliminated. Nonlinear transcendental equations were formulated and solved by analytical algorithms. As mentioned, reliability of the results of these nonlinear equations severely depends on the initial guess, and solutions were not available at some points.

In order to avoid these problems, another method is applied to convert the SHE-PWM problem into an optimization problem. In this method, the objective function is to minimize a quality factor such as total harmonic distortion (THD) or distortion harmonic index (DHI). In this study, THD up to the 49th harmonics order is considered as the objective function. The voltage THD is formulated as

(3) $$\begin{array}{c} \text{THD}\%=\frac{\sqrt{\sum_{n=5,7,\ldots }^{49}a_{n}^{2}}}{a_{1}}\times 100. \end{array}$$

The voltage THD is considered as the objective function *F*(*θ*) in the evolution algorithm. Hence, the minimization problem and its constraints can be represented in a mathematical form as follows:

(4) Min⁡  F(θ)=THD%Sub.:  0<θ1<θ2<⋯<θm<π2;    a1=M;    a5≤ε5;    a7≤ε7;    ai≤εi,

where  *M* is the amplitude of the desired fundamental component,  *ε*_5_, *ε*_7_,…, *ε*_49_ are the allowable limits of individual harmonics mandatory required by Grid Standards [18]. The triple harmonics do not exist in three-phase power system. In this study, a novel PSO is employed to optimize the objective function as described in Section 3.

## 3. Self-Organizing Hierarchical Particle Swarm Optimizer with Time-Varying Acceleration Coefficients

In this section, self-organizing hierarchical particle swarm optimizer with time-varying acceleration coefficient (HPSO-TVAC) is introduced [19] and applied to solve the above harmonic minimization problem. In addition, a comparison with traditional evolution optimizers, such as GA, SFLA, and BA, indicates that HPSO-TVAC gives the best solutions in a wide range of modulation index.

### 3.1. Basic PSO

The basic PSO initiates a random initialization of a population of particles in the search space. Each particle is a potential solution for the optimization problem and tries to search the best position in the total search space. The social behavior of particles that was modeled in the PSO algorithm is used to find the global best solution by simply adjusting each particle's velocity according to its own flying experience and adjusting each particle's position according to the other particles' flying experience.

Each particle is described in the  *d*-dimensional search space by the position vector *X*_*i*_ = [*x*_*i*1_, *x*_*i*2_,…, *x*_*id*_]  and the velocity vector  *V*_*i*_ = [*v*_*i*1_, *v*_*i*2_,…, *v*_*id*_].  In the swarm, the best position of each particle found so far (refer to the optimum fitness function value of each particles) is defined as local best position and is denoted by  *P*_*i*_ = [*p*_*i*1_, *p*_*i*2_,…, *p*_*id*_],  and the global best position of the swarm found so far (refer to the optimum fitness function value of all particles) is denoted by  *P*_*g*_ = [*p*_*g*1_, *p*_*g*2_,…, *p*_*gd*_]. Then, the new velocity of the *i*th particle on the *j*th dimension is updated by using (5) and the position of each particle is updated by using (6):

(5) vij(t+1)=w[vij(t)+φ1r1(pij−xij(t))+φ2r2(pgj−xij(t))],

(6) $$\begin{array}{c} x_{ij}\left(t+1\right)=x_{ij}\left(t\right)+v_{ij}\left(t+1\right), \end{array}$$

where *φ*_1_ and *φ*_2_  are constants known as cogitative and social coefficients, respectively; *r*_1_ and *r*_2_ are random values uniformly distributed within [0,1].  *v*_*ij*_(*t*) is the previous velocity term. The constriction factor *ω*  is used to promise the convergence of the algorithm and defined as

(7) $$\begin{array}{c} w=\frac{2}{\left|2-\varphi -\sqrt{\varphi^{2}-4\varphi }\right|}\;\left(\varphi =\varphi_{1}+\varphi_{2},\;\;\varphi >4\right). \end{array}$$

Typically,  *φ* is set to 4.1, and the constriction factor *ω*  is set to 0.729 [20].

### 3.2. Harmonic Minimization Using HPSO-TVAC

Even though the basic PSO is capable of locating a good solution at a fast rate, its ability to fine tune the global optimum solution is comparatively weak, which is mainly due to lack of diversity at the end of search progress. Thus, HPSO-TVAC is used to solve this problem.

In this novel PSO strategy, the previous velocity term *v*_*ij*_(*t*) in (5) is made zero, and particles rapidly rush towards a local optimum point because of lack of momentum. When a particle stagnates, its local best position  *P*_*i*_  remains unchanged for a number of iterations. When all particles stagnate, the algorithm converges prematurely to a local optimal point and global position *P*_*g*_ remains unchanged and *v*_*ij*_ becomes zero. In order to provide the required momentum for particles to find the global optimum solution in this case, the velocity vector of a particle is reinitialized with a random velocity. In this algorithm, the new velocity of the *i*th particle on the *j*th dimension is updated as follows:

(8) vij(t+1) =[((φ1f−φ1i)iteritermax⁡+φ1i)r1(pij−xij(t))+((φ2f−φ2i)iteritermax⁡+φ2i)r2(pgj−xij(t))],

(9) if  vij=0  and  r3<0.5vij(t+1)=r4×Vmax⁡;elsevij(t+1)=−r5×Vmax⁡;end.

*φ*_1*i*_ and *φ*_1*f*_ are initial and final cogitative coefficients; *φ*_2*i*_ and *φ*_2*f*_ are initial and final social coefficients;  iter and  iter_max⁡_ are current number of iterations and maximum number of iteration;  *r*_1_ ~ *r*_5_  are randomly numbers between [0,1];  *V*_max⁡_ is the maximum velocity value.

**Figure 2:**
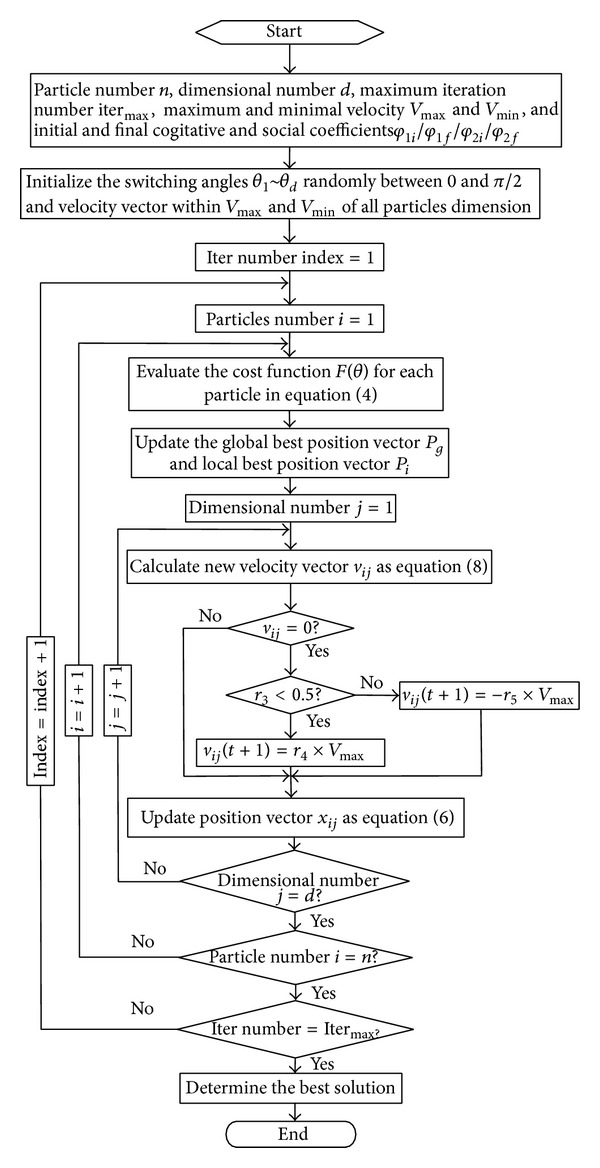
Flow chart of HPSO-TVAC applying to harmonic minimization problem.

**Figure 3:**
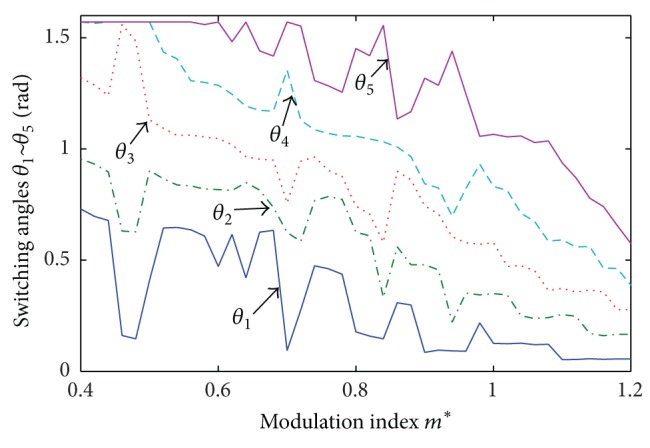
Calculated switching angles  *θ*_1_ ~ *θ*_5_  by HPSO-TVAC.

Thus, a series of particle swarm optimizers are generated inside the main PSO algorithm until the convergence criteria are reached. More details about the HPSO-TVAC were presented in [19]. Appling HPSO-TVAC to the harmonic minimization problem described in above section, the procedure is presented in Figure 2, and the results of optimal switching angles are presented in Figure 3.

### 3.3. Comparison of Traditional Evolution Optimizer

In order to illustrate the efficiency of the applied approach, a comparison between other three evolution optimizers including GA, SFLA, and BA is performed at five operating points of CHB converter. The parameters of these traditional evolution optimizers are set the same as HPSO-TVAC for fair comparison. The operating point is described by the desired modulation index m* which is defined as normalized value of the desired fundamental component.  m*  is given as follows:

(10) $$\begin{array}{c} m^{*}=\frac{M}{sV_{\text{dc}}}. \end{array}$$

Table 1 shows the parameters of HPSO-TVAC and other three evolution optimizers. The number of switching angles is set to five (*s* = 5) and five equal DC sources (*V*_dc_ = 20 V) are considered in the case. The inequality constraint limits (*ε*_5_, *ε*_7_,…, *ε*_49_) in (4) are also shown in Table 1.  Table 2 depicts the calculated switching angles  *θ*_1_ ~ *θ*_5_  and the resulting THD by each method. THD is computed for harmonics up to 49 orders.  Table 3 shows the resulting THD and the variance of THD by each method running 10 times at five operating points (m* = 0.4, 0.5, 0.8, 1.0,1.1).

According to Tables 2 and 3, HPSO-TVAC method produces the smallest THD and the smallest variance of THD in 10 times running. This means that HPSO-TVAC can provide the most stable solution in a wide range of modulation index. In real-time design application, a stable algorithm is important to avoid losing solutions in some operating points and to produce a continuous solution trajectory. The applied approach is more stable and more effective than GA, SFLA, and BA optimizer in harmonic pollution minimization problem.

## 4. Artificial Neural Networks

ANN is a powerful tool to control a nonlinear system that is very complex in nature. A well-designed ANN can be used to replace the look up table and to generate optimal switching angles in a real-time manner [21].

### 4.1. Structure of ANN

ANN consists of a number of fundamental elements named neurons that are organized in several layers. Every neuron has an input vector *P* with *s* inputs. The neuron multiples input vector *P* by a weight matrix  *W* and is summed with a bias vector *b*. Then a transfer function *f* is applied on the above results, which produces the output vector of the neuron *f*(*WP* + *b*) [22, 23].

**Figure 4:**
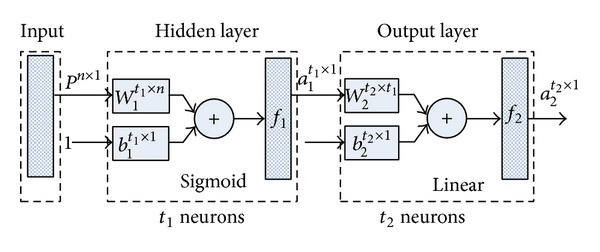
Structure of two-layer feed-forward network.

A typical two-layer feed-forward network shown in Figure 4 with sigmoid hidden neurons and linear output neurons can fit multidimensional mapping problems arbitrarily well. There are *n*  inputs and vectors *P* in an input layer, *t*_1_ sigmoid hidden neurons in hidden layer, and *t*_2_ linear output neurons in output layer. The weight matrix *W*_*i*_ of  *i*th layer with *t*_*i*_ neurons is a *t*_*i*_ × *t*_*i*−1_ matrix. The bias vector *b*_*i*_ of  *i*th layer is a *t*_*i*_ × 1 one and the produced output vector is a *t*_*i*_ × 1 one as well. For the hidden layer, size of *W*_1_ is *t*_1_ × *n*.

The procedure of determination of the weight and bias matrix is named training. In training's iteration process, weight and bias matrices are updated according to previous matrices and a set of desired outputs for specified inputs. Although there are various training algorithms for training neurons network in literatures, the Levenberg-Marquardt optimization (LMO) is chosen as it is almost the fastest back propagation algorithm. The update formulations of weight and bias matrices in LMO are

(11) $$\begin{array}{c} H=J{\left(x\right)}^{T}J\left(x\right),\\ g=J{\left(x\right)}^{T}e\left(x\right),\\ x_{k+1}=x_{k}-{\left[H+\mu I\right]}^{-1}g, \end{array}$$

where *x* is the weight and bias variables matrix,  *e*(*x*) is the error between the desired outputs and the actual outputs of  current network,  *J*(*x*) is the Jacobin matrix that contains the first derivatives of *e*(*x*) with respect to *x*, and  *I*  is the identity matrix. The adaptive parameter  *μ*  is increased by a specified value in [0,1]  until the performance function (mean-squared error) reaches an acceptable value.

The original data set is divided into three subsets: training, validation, and testing. The first subset is for the network training and the network is adjusted to its error. The second subject is used to measure network generalization and to halt training when generalization stops improving. The third subset provides an independent measure of network performance during and after training. The proportion of the data sample adopted in this work is 70% for training, 15% for validation, and 15% for testing.

### 4.2. Implementation of ANN

In order to train ANN, a set of input samples and desired outputs samples are required. In harmonic minimization problem, the inputs are modulation index and the outputs are optimal switching angles obtained by the above HPSO-TVAC. As the modulation index m* value is a continuous variable in [0.4, 1.2], a limited number of m* in this range are sampled. In this paper, only 40 samples are selected in [0.4, 1.2] at 0.02 interval. An ANN with 10 sigmoid neurons in hidden layer and 1 linear neuron in output layer is established to simulate the interpolation fitting problem.

**Figure 5:**
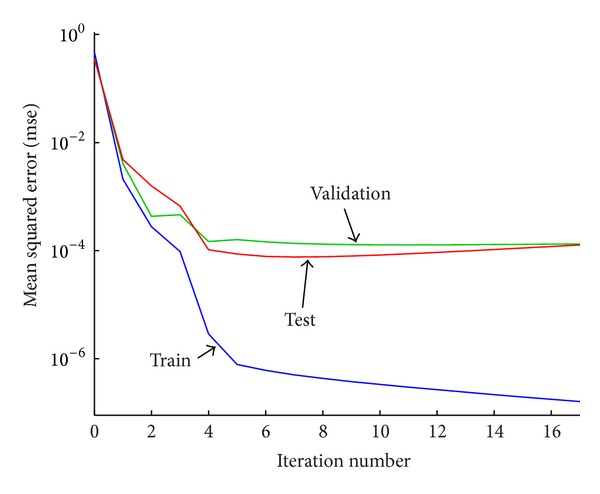
Performance of ANN.

As Figure 5 illustrates, the mean-squared error (MSE) is below 10^−6^ for training, 10^−4^ for validation, and 10^−4^ for testing with 17 iterations. The regression *R* value which measures the correlation between outputs and targets for training, validation, and testing is 0.99997, 0.99996, and 0.9995, respectively.

Table 4 shows the maximum error and the average error between the results generated by ANN and the results calculated by HPSO-TVAC for all angles in different modulation index m*. In any modulation index value in [0.4,1.2], compared with the results using HPSO-TVAC, the maximum and average error for all switching angles using the proposed ANN method are below 10^−3^ and are acceptable. It means that the well-designed ANN trained by 40 samples can produce almost the same optimal switching angles as the HPSO-TVAC method in the whole modulation index range. On one hand, when any DC source value varies, HPSO-TVAC algorithm must be implemented again and new switching angles must be calculated and stored in look-up tables. Look-up tables will increase dramatically when more than one DC source value change. However, the proposed ANN method can adaptively produce the optimal switching angles regardless of DC source values change. This feature is shown in the next section.

On the other hand, the computation time to produce optimal switching angles for a well-designed ANN is about 2 ms, while for HPSO-TVAC it is 0.8 s. Hence a well-trained ANN is a superior substitute for look-up tables in real-time control applications.

### 4.3. Compared with Previous Real-Time Algorithm

**Figure 6:**
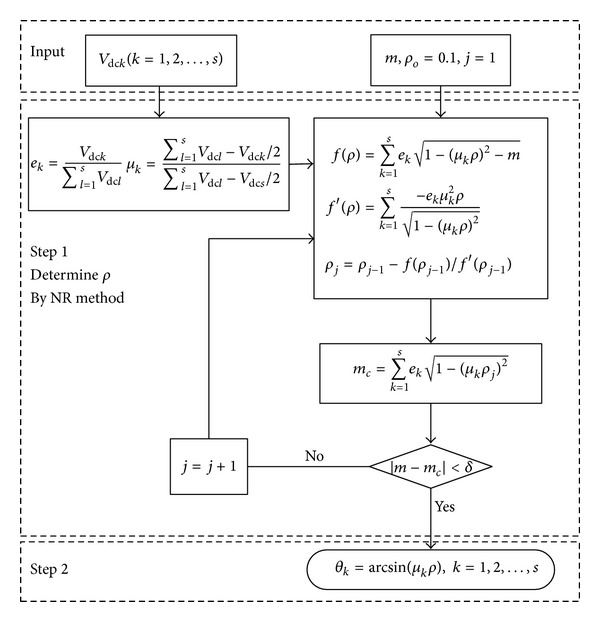
Flow chart of calculation in Yu's paper.

Liu et al. in [15] proposed the first real-time THD minimization algorithm for CHB converter with staircase modulation. The flow chart of this algorithm is shown in Figure 6. This method is based on the Newton-Raphson (N-R) iterative algorithm to minimize the voltage THD. There are two steps in this algorithm.

The first step is to determine the parameter  *ρ* by solving the following equation:

(12) $$\begin{array}{c} \overset{s}{\underset{k=1}{\sum }}e_{k}\sqrt{1-{\left(\mu_{k}\rho \right)}^{2}}=m, \end{array}$$

where *m* is the given modulation index, *s* is the H-bridge number, and DC sources levels are *v*_dc1_ ~ *v*_dc*s*_. Using the N-R iterative algorithm, this nonlinear equation can be solved. In the flow chart, *m*_*c*_ is a calculated modulation index during the iteration. If the difference between *m*_*c*_ and *m* is small enough (<*δ*), the iteration will end and the resulting THD will be minimum. *δ* is the threshold value 10^−5^.

The second step is to output the optimal switching angles  *θ*_1_ ~ *θ*_*s*_:

(13) $$\begin{array}{c} \theta_{k}=\text{arc}\sin\left(\mu_{k}\rho \right),\;k=1,2,\ldots ,s. \end{array}$$

**Figure 7:**
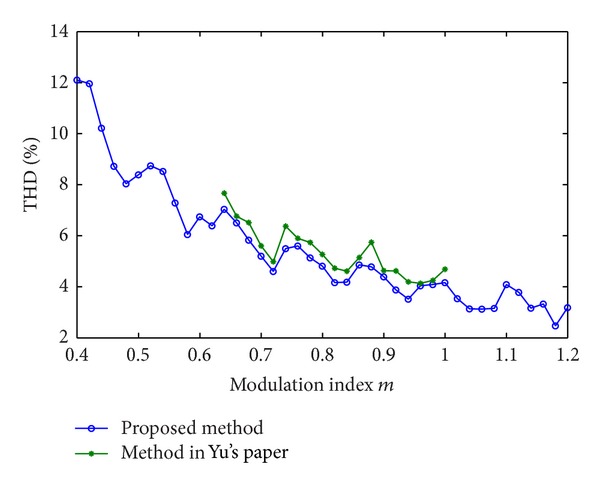
Comparison of THD between the proposed method and the previous method.

**Figure 8:**
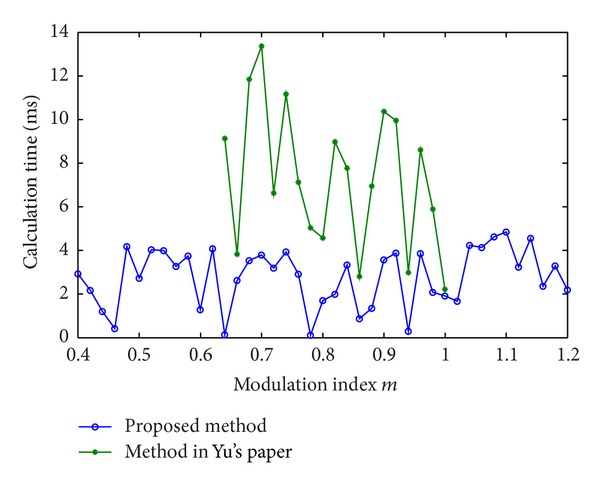
Comparison of calculation time between the proposed method and the previous method.

Taking the above five-stage 11-level CHB converter (*s* = 5) as an example, the calculated THD of the proposed method and Yu's method is compared and shown in Figure 7. The calculation time determined by the proposed method and Yu's method is also compared and shown in Figure 8.

It can be seen that the previous real-time algorithm produces almost the same optimal switching pulses and voltage THD value as the proposed method does. However, the previous method is only suitable for a small range of modulation index ([0.65–0.98]). The proposed algorithm is suitable for a wider range of modulation index ([0.4–1.2]). In addition, the proposed method results in a smaller THD and a lower calculation time, compared with the previous one. The reason is that the N-R iterative algorithm is inherently sensitive to initial values. It may lose solutions in some operating points and cost more time to search for solutions in the iteration process.

Table 5 shows the cost time and memory for several methods in one running. The traditional method must store solutions for every possible operating point and the resulting memory is very large. The proposed method is adaptive and the needed memory is small. Therefore, from the point of view of the voltage THD and real-time implementation, the proposed method is better than the previous method.

## 5. The Proposed Closed-Loop Algorithm

The closed-loop control algorithm for three-phase CHB converter is always designed at high switching frequency (>5 k) with PSPWM or SVPWM modulation strategy. Although the classical two-loop control structure with PI controller has good dynamic performance, this high frequency method suffers high switching loss and requires many strictly equal DC sources. In this paper, a closed-loop control algorithm based on ANN for CHB converter is proposed.

### 5.1. Closed-Loop Control Algorithm Based on ANN

**Figure 9:**
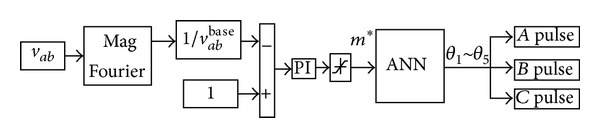
The proposed closed-loop control algorithm.

As shown in Figure 1(a), the three-phase load is balanced and the load line-voltage *v*_*ab*_ is sampled to keep constant load voltage. The block diagram of proposed closed-loop control algorithm is shown in Figure 9.

A Fourier block is used to calculate the fundamental component in load line-voltage *v*_*ab*_.  The error between the desired line-voltage and the calculated one is fed to a simple PI controller. The PI controller is used to adjust the modulation index  m* and ANN is used to generate optimal switching angles *θ*_1_ ~ *θ*_5_ online. Pulses block is used to generate switching signals to driven H-bridges. In order to investigate the proposed closed-loop control algorithm, a test bench shown in Figure 1(a) is constructed in MATLAB/Simulink software.

**Figure 10:**
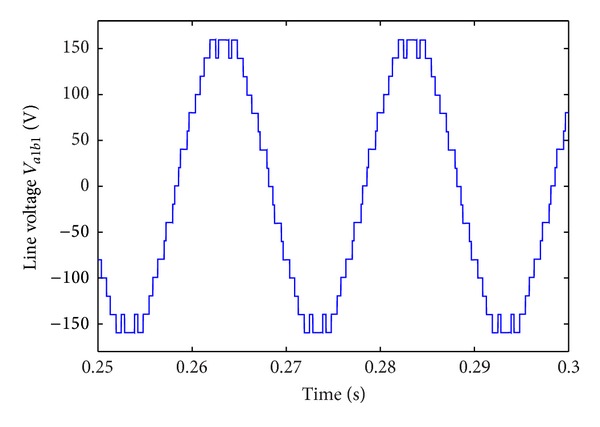
The converter's output line-voltage  *V*_*a*_1_*b*_1__.

**Figure 11:**
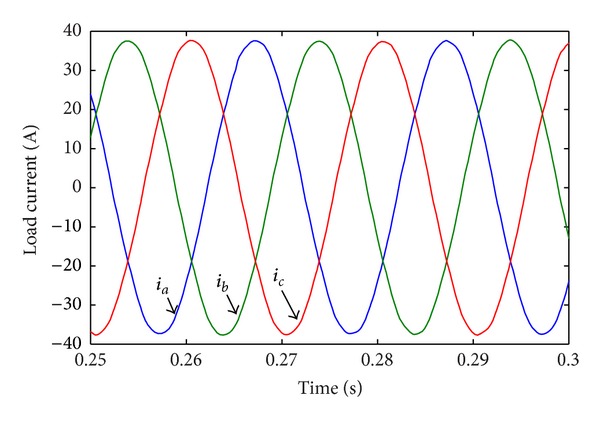
Three-phase load current  *i*_*abc*_.

Table 6 shows the 11-level CHB converter's parameters.  Figure 10 shows the CHB converter's steady state output line-voltage  *V*_*a*_1_*b*_1__.  Figure 11 shows the three-phase load current. Harmonic analysis shows that THD of the load current is small (0.81%). The above results indicate that the proposed algorithm is effective to minimize load current's THD.

### 5.2. Dynamic Analysis

In real-time control applications, closed-loop control algorithm must be used to quickly stabilize load voltage and avoid current pollution when subjecting to DC sources disturbance or load disturbance. In this study, two cases are used to test the performance of the proposed closed-loop control algorithm. The proportional gain in PI controller is 1 and integral gain is 100. The sampling frequency is 10 kHz.

**Figure 12:**
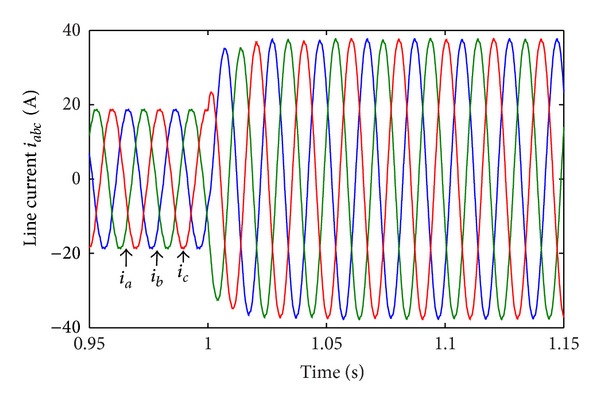
Three-phase load current response to load disturbance.

**Figure 13:**
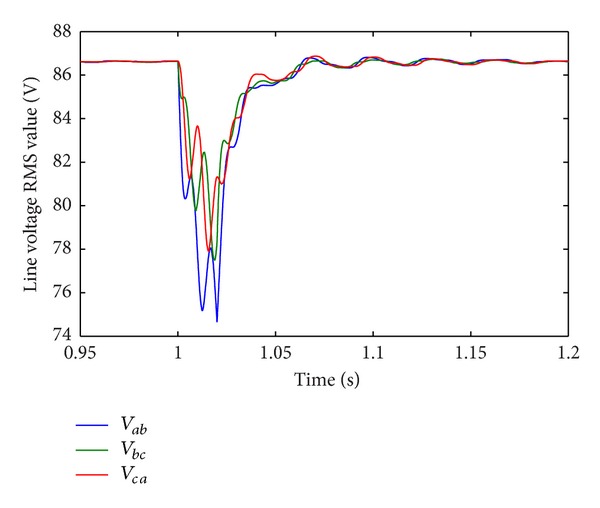
RMS of line-voltage response to load disturbance.

**Figure 14:**
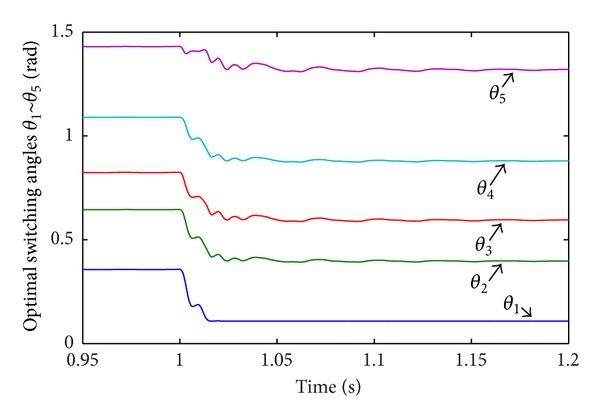
Optimal switching angles response to load disturbance.

In Case 1, load disturbance is applied on CHB converter. The load steps form half load to full load at  *t* = 1.0 s.  Figure 12 shows the corresponding line-current response. The THD of load current before and after the disturbance is 2.21% and 0.81%.  Figure 13 shows the corresponding RMS of line-voltage response.  Figure 14 shows the corresponding optimal switching angles response. The transient recovery time is about 0.2 s.

**Figure 15:**
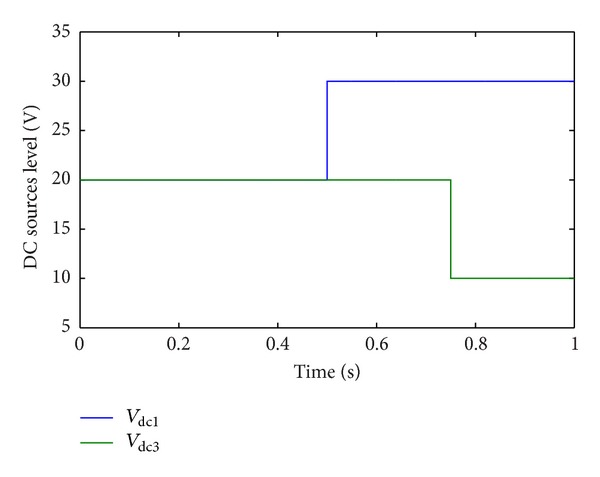
DC sources disturbance.

**Figure 16:**
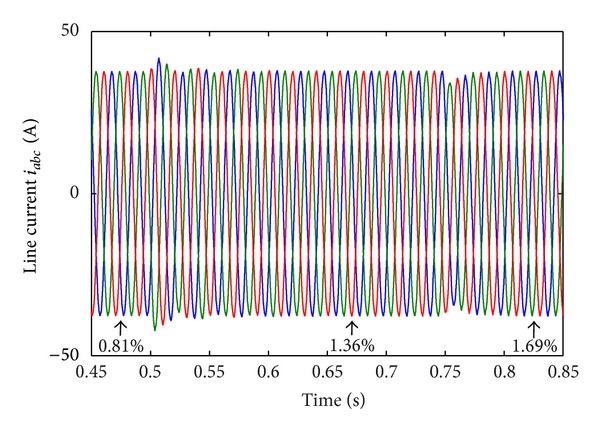
Three-phase load current response to DC sources disturbance.

**Figure 17:**
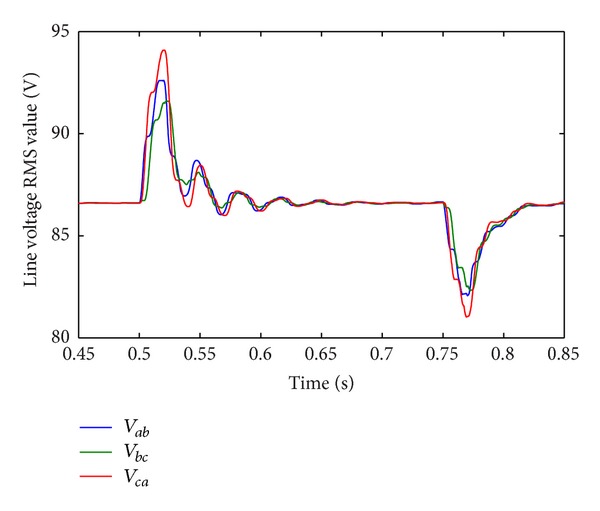
RMS of line-voltage response to DC sources disturbance.

**Figure 18:**
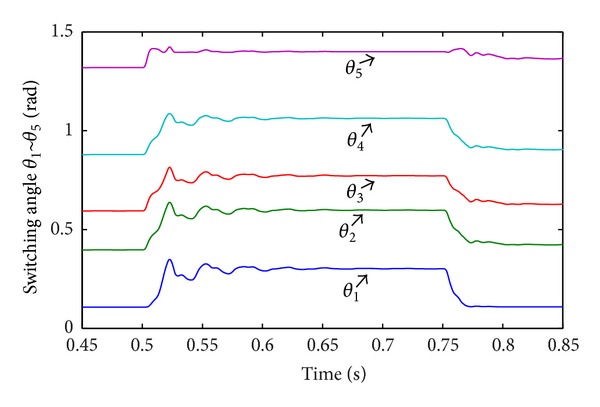
Optimal switching angles response to DC sources disturbance.

In Case 2, DC sources disturbance is applied on CHB converter. The first DC voltage level *v*_dc1_ in each phase changes from 20 V to 30 V at  *t* = 0.5 s. The third DC voltage level *v*_dc3_ in each phase changes from 20 V to 10 V at *t* = 0.75 s.  Figure 15 shows the DC voltage disturbance.  Figure 16 shows the corresponding line-current response. Although the multilevel structure is considered asymmetric in this condition, the current is still balanced and has a low THD value (1.69%).  Figure 17 shows the corresponding RMS of line-voltage response.  Figure 18 shows corresponding optimal switching angles response.

The above results show that the proposed closed-loop control algorithm is effective to stabilize load voltage and minimize the line current's THD when subjecting to dynamic disturbance, such as DC sources disturbance and load disturbance. Especially, when the multilevel structure is considered asymmetric, the current is still balanced and avoids harmonic pollution. The CHB converter does not need many strictly equal DC sources by this algorithm, avoiding the drawbacks of traditional PSPWM or SVPWM control algorithm. The proposed closed-loop control algorithm has good control capability of CHB converter.

### 5.3. Real Design Issue

In real design stage, hardware implementation issue of RBF neural network and switching angles pulse generation must be considered. As the modern DSP and FPGA device's computation capability and memory increase, RBF neural network can be implemented on embedded systems. Lots of technique reports proposed various methods to address this issue [24, 25]. Thus, this section considers only the switching angles pulse generation issue.

**Figure 19:**
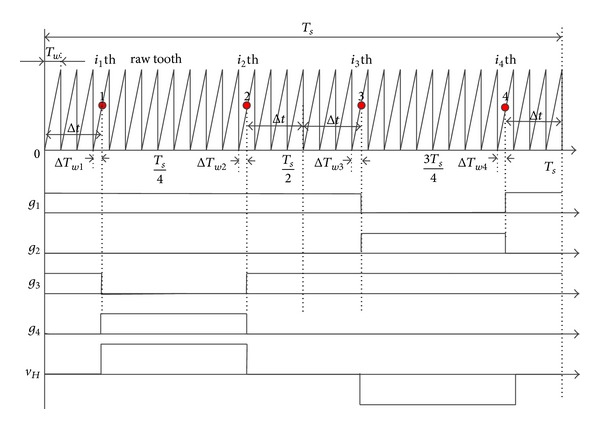
Proposed pulse generation scheme according to the switching angles.

The pulse generation can be implemented by TI DSP 28335's ePWM module. The time-based counter produces a saw-tooth waveform with a period  *T*_*w*_. The voltage's fundamental period is  *T*_*s*_. The switching angle *θ* is represented by an interval Δ*t*. The corresponding switching signal  *g*_1_ ~ *g*_4_  for four power switches  *S*_1_–*S*_4_  in one H-bridge of CHB converter can be generated as shown in Figure 19. The H-bridge's output voltage is  *v*_*H*_. The high voltage level in *g*_*i*_  (*i* = 1, 2, 3, 4) means that the corresponding switch  *S*_*i*_ is turned on and low voltage level means that the corresponding switch *S*_*i*_  is turned off.

The turn-off time and the turn-on time for *S*_1_ are located at *i*_3_th and *i*_4_th saw-tooth in interval *T*_*s*_, respectively. They can be determined by Δ*T*_*w*3_  and Δ*T*_*w*4_  given as follows:

(14) $$\begin{array}{c} \Delta t=\frac{\theta }{2\pi }T_{s},\\ i_{3}=\text{Round}\left[\frac{\left(T_{s}/2+\Delta t\right)}{T_{w}}\right],\\ \Delta T_{w3}=\left(\frac{T_{s}}{2}+\Delta t\right)-i_{3}\times T_{w},\\ i_{4}=\text{Round}\left[\frac{\left(T_{s}-\Delta t\right)}{T_{w}}\right],\\ \Delta T_{w4}=\left(T_{s}-\Delta t\right)-i_{4}\times T_{w}. \end{array}$$

The turn-off time and turn-on time for *S*_3_ are located at *i*_1_th and *i*_2_th saw-tooth in interval *T*_*s*_, respectively. They can be determined by Δ*T*_*w*1_ and Δ*T*_*w*2_ given as follows:

(15) $$\begin{array}{c} i_{1}=\text{Round}\left[\frac{\Delta t}{T_{w}}\right],\;\;\Delta T_{w1}=\Delta t-i_{1}\times T_{w},\\ i_{2}=\text{Round}\left[\frac{\left(T_{s}/2-\Delta t\right)}{T_{w}}\right],\;\;\\ \Delta T_{w2}=\left(\frac{T_{s}}{2}-\Delta t\right)-i_{2}\times T_{w}. \end{array}$$

The signals, *g*_1_ and *g*_2_, *g*_3_ and *g*_4_, have opposite polarity to avoid simultaneous conduction of the upper and lower switch. As shown in Figure 19, the high level width in *v*_*H*_  is directly related to switching angle *θ*. Thus, five different width pulse voltages can synthesize the ac terminal output phase voltage *v*_an_ shown in Figure 1(b). When the saw-tooth waveform's period *T*_*w*_ is smaller, the pulse generation is more accurate.

**Figure 20:**
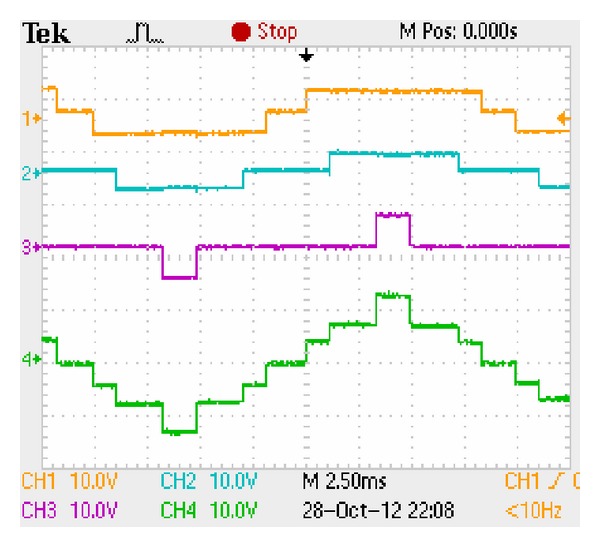
Three-stage 7-level staircase waveform.

**Figure 21:**
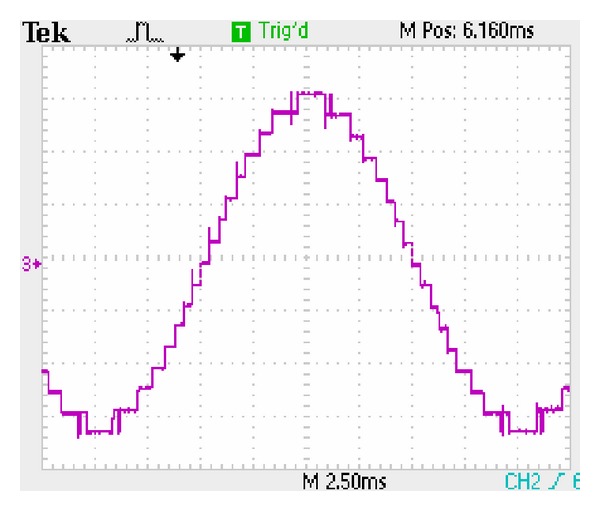
Five-stage 11-level staircase waveform.

The three-stage 7-level CHB converter's experimental output phase voltage is shown in Figure 20. The five-stage 11-level CHB converter's experimental output phase voltage is shown in Figure 21.  *T*_*s*_  is 0.02 s for 50 Hz signal. *T*_*w*_ is 20 *μ*s. Although pulses generation principle for only one operating point is given, the switching angles for other operating points can be generated similarly. The experimental results verify the proposed pulse generation scheme.

## 6. Conclusion

The contribution of this paper is that a real-time, closed-loop control algorithm based on ANN for three-phase CHB inverter is proposed. It costs little computation time and memory to generate optimal switching angles and has a good control capability of CHB converter. Compared with the previous real-time optimal switching angles method, the proposed one needs little computation time and can produce better solutions in a wide modulation index [0.4–1.2]. Moreover, the proposed algorithm is closed-loop and able to quickly stabilize load voltage and minimize the line current's THD subjecting to DC sources disturbance and load disturbance. In addition, a switching angle pulse generation scheme is proposed and experiment results verify its correctness.

## Figures and Tables

**Table 1:** Parameters of evolution optimizers.

Parameters	HPSO-TVAC	GA	SFLA	BA
Population size	100	100	100	100
Iteration number	500	500	500	500
Number of runs	10	10	10	10
*φ*_1*i*_/*φ*_1*f*_/*φ*_2*i*_/*φ*_2*f*_	2.5/0.2/0.2/2.2			
*ε*_5_/*ε*_7_ ⋯ /*ε*_49_/%	5/4/3/⋯/0.2 + 25/*h*			
Modulation index rang	0.4–1.2			
Calculation step of m*	0.01			

**Table 2:** Calculated switching angles by different methods at operating point (m* = 1.0).

Method	*θ*_1_ */*rad	*θ*_2_/rad	*θ*_3_/rad	*θ*_4_/rad	*θ*_5_/rad	THD%
HPSO	0.1256	0.3495	0.5788	0.8310	1.0655	4.17
GA	0.1269	0.3455	0.5738	0.8192	1.0799	4.32
SFLA	0.1352	0.3476	0.5731	0.8336	1.0648	4.26
BA	0.1238	0.3480	0.8345	0.5785	1.0636	4.18

**Table 3:** THD results and variance of THD in 10 times running at five operating points.

m* THD%/variance	GA	BA	SFLA	HPSO-TVAC
0.4	12.28/0.03	12.15/0.06	12.11/0.02	12.10/0.02
0.6	6.61/0.03	5.89/0.06	6.70/0.02	5.78/0.01
0.8	5.39/0.04	4.93/0.05	4.83/0.01	4.82/0.01
1.0	4.32/0.04	4.18/0.07	4.26/0.03	4.17/0.02
1.1	4.22/0.04	4.19/0.07	4.22/0.02	4.09/0.01

**Table 4:** Maximum and average of errors of ANN results for all angles.

Switching angles/rad	*θ*_1_	*θ*_2_	*θ*_3_	*θ*_4_	*θ*_5_
Maximum error/1*E* − 3	3.4	8.9	5.6	1.2	0.9
Average error/1*E* − 4	5.2	5.1	4.8	7.4	9.6

**Table 5:** Comparison of real-time algorithms.

Algorithm	Memory^17^	Cost time
N-R	25 kB	1 s
Evolution algorithm	25 kB	0.8 s
ANN	200 B	2 ms

**Table 6:** Parameters about 11-level CHB converter.

Power	4 kW
Filter *L*_*a*_/*L*_*b*_/*L*_*c*_	4.7 mH
Load	1.875 *Ω*
DC voltage	20 V
Stage number	5
